# Syringopleural shunt placement in a pug with a cervical spinal diverticulum and associated syringomyelia

**DOI:** 10.1002/ccr3.2845

**Published:** 2020-04-13

**Authors:** Anna Tauro, Clare Rusbridge

**Affiliations:** ^1^ ChesterGates Veterinary Specialists Chester UK; ^2^ School of Veterinary Medicine Guildford UK; ^3^ Fitzpatrick Referrals Eashing UK

**Keywords:** computed tomography, corticosteroid, laminectomy, magnetic resonance imaging, myelopathy, tetraparesis

## Abstract

We report persistence of associated syringomyelia and formation of newly caudal spinal arachnoid diverticulum, following marsupialization surgery. We describe syringopleural shunt placement as a novel approach to treat both conditions in a Pug dog.

## Graphical abstract

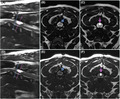

## 1 INTRODUCTION

A 6‐month old male entire Pug dog with a 2‐month history of ataxia was referred to Fitzpatrick Referrals Neurology service for further investigations. On presentation, the dog's mentation was normal; he showed ambulatory tetraparesis and proprioceptive ataxia. Overreaching or floating thoracic limb gait was noted (Video S1) with a tendency for toenail scuffing and evidence of skin abrasions on the dorsal surface of the feet, mainly of his left thoracic limb. Postural reactions were reduced in all limbs, with his left side being slightly worse. Spinal reflexes were intact, and cranial nerve assessment was unremarkable. There was no evidence of discomfort, and no treatment was prescribed by his veterinarian prior referral. The dog showed a concurrent and mild brachycephalic obstructive airway syndrome (BOAS). The neuroanatomic localization was determined to be C1‐C5 spinal cord segments.

A complete blood cell count, serum biochemistry, and electrolytes were performed and were unremarkable.

## 2 CASE SUMMARY

Magnetic resonance (MR) images of the brain and cervical spine were acquired under general anesthesia using a 1.5 T scanner (Tim system, Siemens). The dog was positioned head first in dorsal recumbency on the scanning table, and MR data were obtained using a combination of the spine, neck, and body and spinal coils.

The brain protocol included T2‐weighted (T2W; TR = 3590 ms, TE 109 ms, FOV 105 × 140, thickness = 3.0 mm, spacing = 0.6 mm, matrix = 239 × 384 and NEX 2) and T1‐weighted (T1W; TR = 515 ms, TE 14 ms, FOV 112 × 140, thickness = 3.0 mm, spacing = 0.6 mm, matrix = 192 × 320 and NEX 3) in transverse planes.

The spinal protocol included T2W in sagittal (T2W; TR = 4000 ms, TE 105 ms, FOV 220 × 220, thickness = 1.5 mm, spacing = 0.3 mm, matrix = 358 × 448 and NEX 4) and transverse planes (T2W; TR = 3480 ms, TE 101 ms, FOV 91 × 140, thickness = 3 mm, spacing = 0.3 mm, matrix = 164 × 384 and NEX 5), STIR in dorsal plane (STIR; TR = 3200 ms, TE 50 ms, FOV 160 × 270, thickness = 3 mm, spacing = 0.6 mm, matrix = 148 × 384 and NEX 2), and 3D‐CISS sequence in transverse plane (3D‐CISS; TR = 11.72 ms, TE 5.86 ms, FOV 93 × 150, thickness = 1.0 mm, spacing = 0.2 mm, matrix = 160 × 256 and NEX 1). With the aid of the multiplanar reconstruction software integrated to the MRI scanner, 3D‐CISS data were further processed in sagittal and dorsal planes.

Computed tomography of the head, neck, and thorax was performed under general anesthesia to evaluate the correct shunt placement (Aquilion PRIME, Toshiba Medical Systems, 160‐slice multislice CT scanner). The dog was positioned head first in sternal recumbency on the scanning table, and images were acquired using a helical scan with 0.5‐mm slice thickness and reconstructed in bone and soft tissue algorithms as 2.0‐mm transverse images and 0.5‐mm multiplanar images (120 kV, 34‐65 mAs, 240 mm FOV).

**Figure 1:**
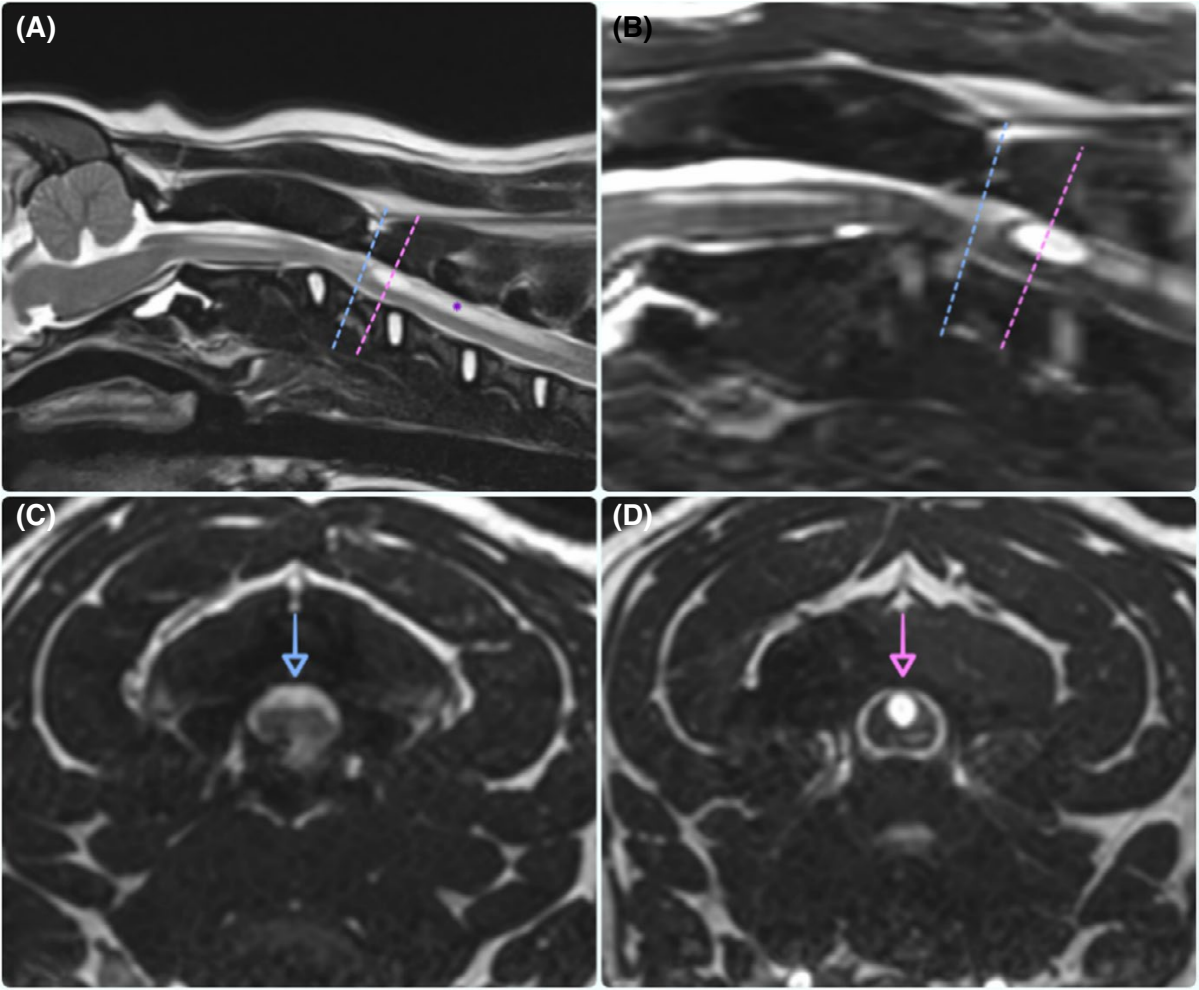
Magnetic resonance images; mid‐sagittal T2W (A), 3D‐CISS (B), and transverse 3D‐CISS (C and D) images at the level of the C2‐C3 intervertebral disc space showing the presence of spinal arachnoid diverticulum (SAD) (blue dashed line and arrow) and syringomyelia (SM) (pink dashed line and arrow)

The MR images of the brain showed a mild ventriculomegaly and a supracollicular fluid accumulation, which were considered nonclinically significant.^1^, ^2^ The MR images of the cervical spinal cord showed the presence of a dorsal intradural‐extramedullary cavity filled with CSF supporting the presence of a spinal arachnoid diverticulum (SAD) (L8mm × H2mm × W6mm) at the level of C2‐C3 intervertebral disc (IVD) space with secondary spinal cord compression. An hyperintense on T2WI and 3D‐CISS and hypointense on T1WI structure compatible with syringomyelia (SM) (L7.3mm × H3.3mm × W3mm) were present centrally and within the dorsal half of the spinal cord, just caudal to the SAD (Figure 1). Central canal dilation (transverse diameter: 1.2 mm) was present over the length of the C2 vertebra, while an intramedullary hyperintensity on T2WI and isointensity on T1WI area compatible with spinal cord edema were visible over C4‐C5 vertebrae.

The MR findings supported the presence of SAD, while SM was considered secondary to SAD due to cerebrospinal fluid (CSF) flow obstruction. The dog underwent surgery to address SAD via left dorsolateral laminectomy at the level of C2‐C3 IVD space. A surgical scalpel blade no. 11 with bevel pointing upward was used to perform a small durotomy through which 45° angled microscissors were placed to extend the durotomy, followed by marsupialization of the diverticulum using 6‐0 Vicryl™ (Ethicon™) absorbable braided suture, anchoring the dural margins to the surrounding epaxial muscles. The dog received anti‐inflammatory dose of prednisolone (Table 1) perioperatively to reduce the spinal cord edema. Multimodal analgesia with methadone at the dose of 0.2 mg/Kg IV every 4‐6 hours (Comfortan™ 10 mg/mL, Dechra) and paracetamol at the dose of 10 mg/Kg PO twice daily (Paracetamol suspension 120 mg/5 mL, McNeil) was also provided over the postoperative period.

**Table 1:** Timeline of the clinical signs, imaging performed, and corticosteroid therapy

Timeline	Age	Prednisolone therapy	Comments
First manifestation of neurological signs	4 mo		
First referral consult& MRI & marsupialization	6 mo	Perioperative.	Diagnosis of SAD and SM was made. 0.3 mg/Kg SID for 7 d, 0.15 mg/kg SID for 10 d, and 0.15 mg/kg EOD.
		Restarted approx. 3 wk postsurgery.	The dog improved at the discharge but worsened a week after discontinuation of prednisolone, showing increased scuffing on thoracic limbs and falling onto his right side. Amelioration was reported after initiation of corticosteroids at 0.3mg/Kg SID.
2nd MRI (2 mo postsurgery)	8 mo	Continued.	SAD resolved, but SM was still present. Spinal cord edema increased, extending over C4‐C6 vertebrae. Newly formed SAD noted caudal to the previous surgical site. Shunt kit was ordered.
	10 d post‐MRI	Dose increased.	Scuffing and bleeding from dorsal aspect of front paws. Increased corticosteroids to 0.4 mg/kg SID.
	2 wk post‐MRI	Continued but dose slightly reduced.	Failed to improve with medical therapy and worsening of thoracic limb paresis with tendency for toenail scuffing. Reported possible sign of hyperaesthesia related to SM (ie, licking at his paws). Gabapentin added at 15 mg/kg BID. Corticosteroid dose was slowly tapered down, but not able to lower below 0.3 mg/Kg SID, without deterioration.
3rd MRI (6 mo postsurgery)	1 y	Continued.	SAD and SM remained unchanged, although spinal cord edema increased extending over C4‐C7 vertebrae.
Shunt surgery	1 y 2 mo	Continued.	Prior surgery, rubbing face on furniture indicating hyperesthesia: increased gabapentin to TID. The dog showed amelioration of signs.
Recheck	1 y 4 mo	Continued.	Ataxia and postural reactions have improved with his right thoracic limb remaining slightly paretic; hypermetria on thoracic limbs was still present.
Recheck &CT & revision surgery	1 y 5 mo	Continued.	Worsening of ataxia with knuckling and falling over onto his right side. On CT, distal shunt catheter dislodged. Shunt revision performed.
Recheck	1 y 6 mo	Continued.	Remained stable with no overall improvement. Starting to use quad wheelchair as aid to ambulate.
4th MRI (6‐mo after shunt surgery)	1 y 8 mo	Continued.	On MRI, resolution of previous SAD and SM, but newly formed SAD noted. Mild spinal cord edema over C4 vertebra. Neurologically stable with mild knuckling on his right thoracic limb.
	2‐2.5 y	Reduced.	Recurrence of face rubbing and left pelvic limb tremors was reported; signs resolved when gabapentin was restarted at 15 mg/kg BID for a week. Gradually reduced steroids to 0.15 mg/kg EOD.
Phone call update (2 y postshunt surgery)	3 y 2 mo	Off.	Off steroids. Reported recurrence of licking paw and rubbing face which resolved with hydrotherapy. Slight thoracic limb hypermetria and ataxia present, but overall stronger on all limbs.

Abbreviations: BID, twice daily; CT, computed tomography; EOD, every other day; MRI, magnetic resonance imaging; SAD, spinal arachnoid diverticulum; SID, once daily; SM, syringomyelia; TID, three times daily.

The dog underwent physiotherapy in order to support his recovery. Prednisolone was restarted approximately 3 weeks after surgery, as signs of ataxia and stumbling of his thoracic limbs worsened. The neurological deficits appeared to be slightly worse on his right side. The dog showed a slight amelioration of his clinical signs while receiving corticosteroids.

**Figure 2:**
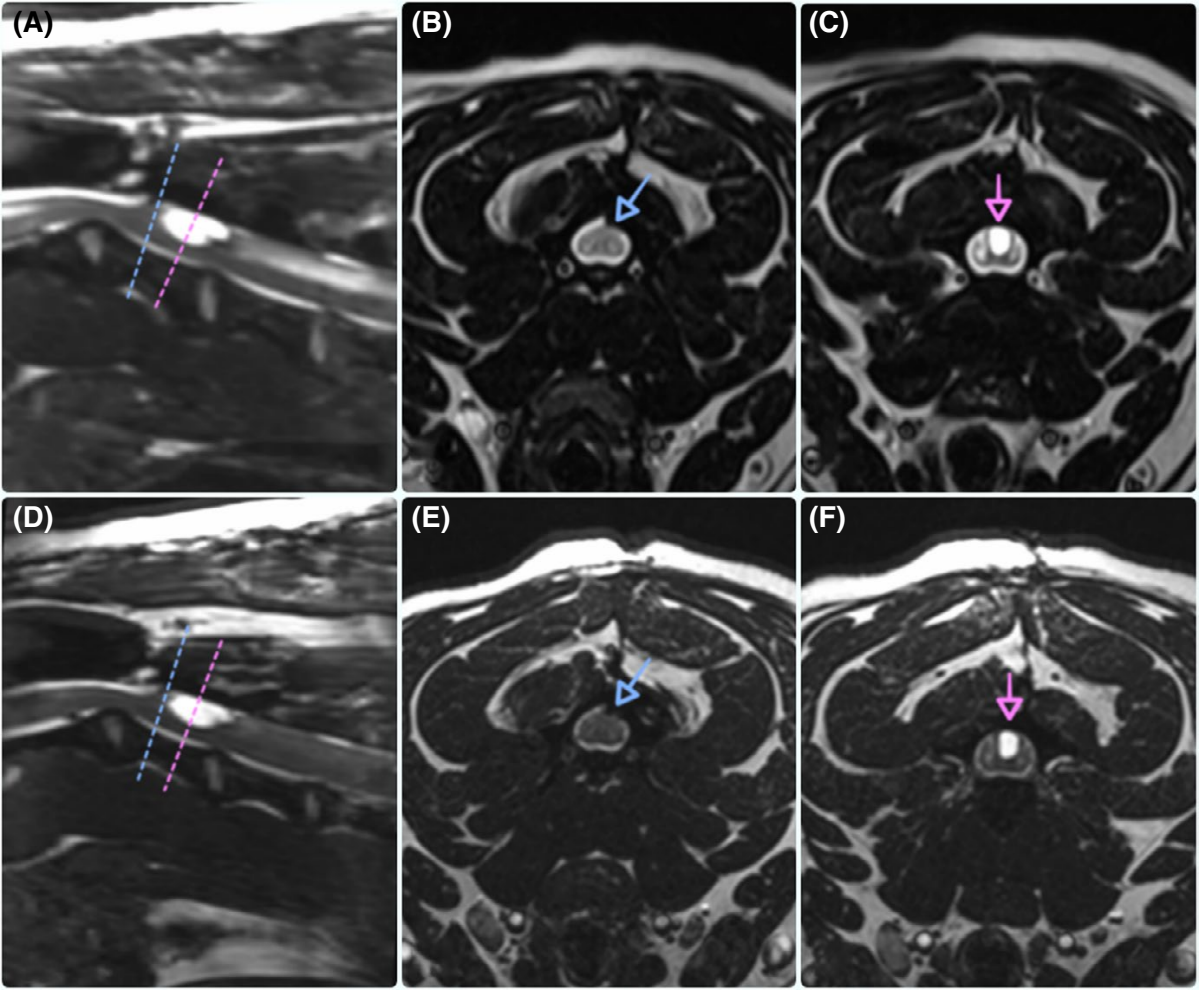
Magnetic resonance images; mid‐sagittal 3D‐CISS (A and D) and transverse 3D‐CISS (B, C, E, F) images at the level of the C2‐C3 intervertebral disc space showing the laminectomy site (blue dashed line and arrow) and syringomyelia (SM) (pink dashed line and arrow) at 2m (A, B, C) and 6m (D, E, F) postsurgery to address SAD

MRI of the cervical spine was performed at 2 (Figure 2A‐C) and 6 months (Figure 2D‐F) postsurgery and revealed the resolution of the previous SAD; however, a newly formed CSF‐filled structure (L9.5mm × H2.5mm × W3mm) was found dorsally to the SM and caudally to the surgical site. Spinal cord edema progressively worsened, extending over C4‐C6 and over C4‐C7 vertebrae at 2 months and 6 months postsurgery, respectively. Further neurological deterioration was reported, with increased thoracic limb paresis and tendency for toenail scuffing causing skin excoriation on the dorsal surface of the feet. Signs of possible hyperaesthesia secondary to SM (ie, licking at his paws) were also present.^3^ Prednisolone was increased to approx. 0.4 mg/Kg once daily, and gabapentin was started at the dose of 15 mg/Kg twice daily. Prednisolone was tapered to 0.3 mg/Kg once daily, as lower dose caused further deterioration. Prior to shunt surgery, the dog started to rub his face on furniture (ie, hyperesthesia). Gabapentin was increased from twice to three times daily, and the signs appear to subside. A syringopleural shunt was placed approximately 6 months after the newly formed SAD was diagnosed, due to the delay of delivery of the shunt kit. Surgical approach at the level of the C2‐C3 IVD space was performed, and the previous laminectomy site was identified: Laminectomy membrane was not present; however, scar tissue was found adhering to the dura mater. An attempt to remove this was made but abandoned, due to the multiple attachments and the unlikeliness of success. Dorsal laminectomy was extended caudally over C3 vertebra until the newly CSF‐filled structure was identified by bluish discoloration of the dura mater. The perforated, open‐ended proximal tip of the catheter was trimmed to match the length of the CSF‐filled cavity (approximately 0.5 mm) based on CT measurement and angled‐cut for easy insertion. A limited durotomy, so that the shunt catheter will just pass through the opening, was performed using a surgical scalpel blade no. 11 with bevel pointing upward. CSF outflow from the incised dura confirmed the correct location, and the proximal tip of the syringopleural shunt was placed through the dural incision using a CODMAN™ lumboperitoneal catheter, JAMES™ design (internal diameter 0.76 mm; external diameter 1.65 mm), inserted at the level of the C3 and in a cranial direction. The presence of CSF flowing through the distal catheter tip ensured its correct placement and functionality. Pressure was controlled through distal slit valves (5‐9cm H_2_O medium pressure), four slits 90° apart. The incised dura was anchored over the shunt catheter using single interrupted 6‐0 Vicryl™ (Ethicon™) absorbable braided suture, the proximal catheter was subsequently looped three times and at each bend, and purse‐string sutures were applied using a synthetic, monofilament, nonabsorbable polypropylene suture (Prolene™ 5‐0) to anchor the catheter to the surrounding epaxial muscles. The dural incision did not need further closure, as no CSF leakage was present around the shunt catheter. An absorbable collagen fleece (Lyostypt™) was placed over the laminectomy site. The distal tip of the shunt catheter was inserted subcutaneously and passed tangentially across the intercostal muscles into the pleural space at the left ninth intercostal space. Two concentric nonabsorbable polypropylene pursue‐string sutures (Prolene™ 5‐0) were applied anchoring the tube to the intercostal muscles. The ends of the sutures were used to perform a "Chinese‐finger trap" suture around the distal catheter. Skin closure was performed using a nonabsorbable monofilament (Ethilon™ 3‐0) single interrupted cruciate sutures. Postoperative CT confirmed the correct placement of the shunt and the resolution of the CSF‐filled structure. Over the following 2 months, the patient continued the physiotherapy and showed a progressive neurological amelioration, although a mild ataxia, thoracic limb hypermetria, and right‐sided paresis persisted. Despite several attempting in reducing the dosage, prednisolone was continued as previously stated (Table 1).

Three months after shunting, the dog was re‐examined due to an acute collapse episode. CT was performed and showed the resolution of both SAD and SM; however, the distal tip of the shunt catheter was displaced and coiled subcutaneously at the level of its insertion into the pleural space. Revision surgery was performed, and the shunt catheter was re‐inserted into the pleural space. The patency of the catheter was confirmed intra‐operatively with the presence of CSF‐like fluid flow at the distal tip. Postoperative CT was performed to confirm the correct shunt placement.

At 1‐month re‐examination, the dog's neurological status was unchanged and continued to show mild ataxia and right‐sided paresis with the tendency to stumble on his thoracic limbs; prednisolone was continued. Hydrotherapy and a quad wheelchair for dogs (Walkin' Wheels^®^) were provided to support his recovery.

**Figure 3:**
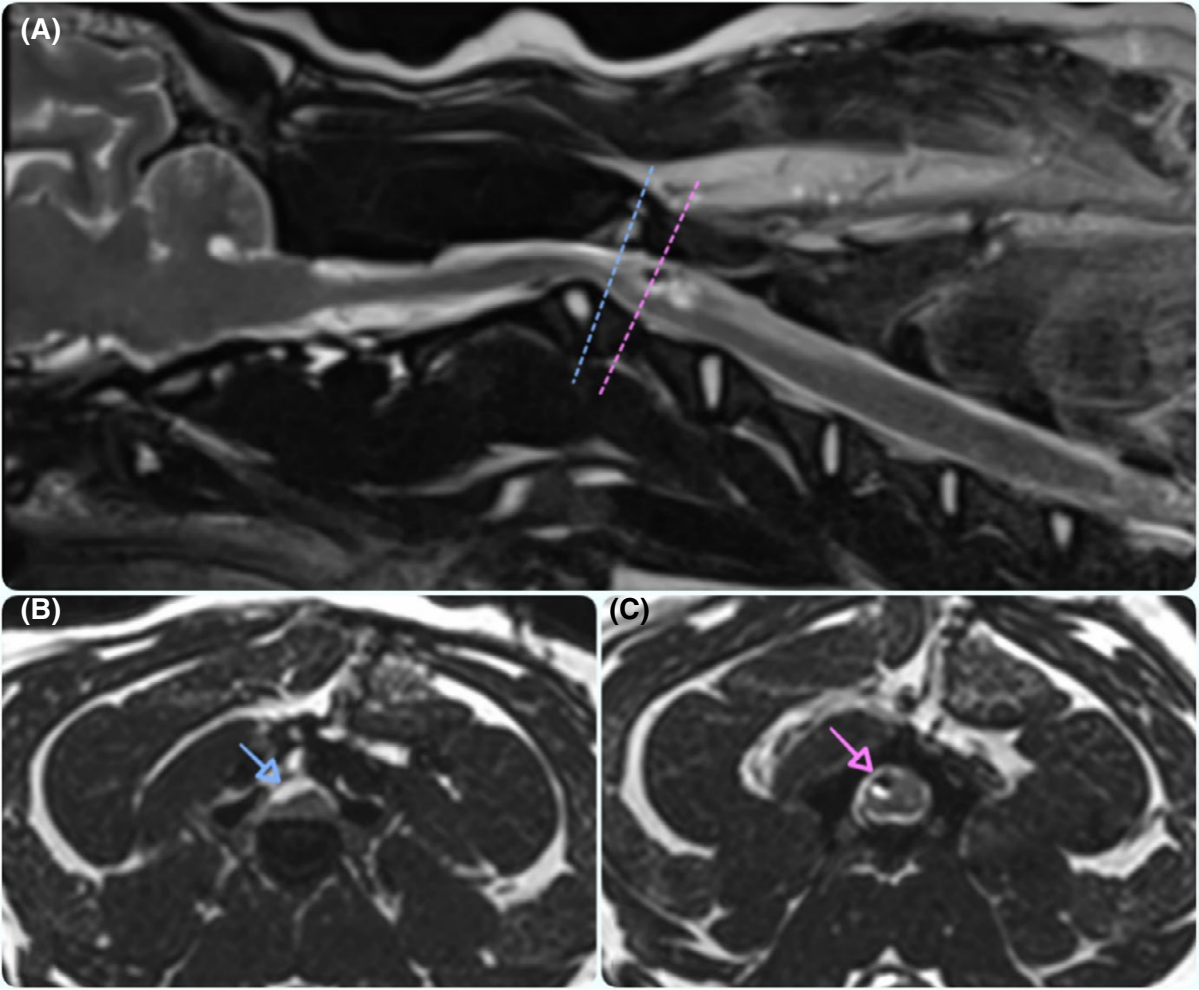
Magnetic resonance images; para‐sagittal 3D‐CISS (A) and transverse (B and C) images at the level of the C2‐C3 intervertebral disc space showing a newly formed spinal arachnoid diverticulum (SAD) (blue dashed line and arrow) on the right side and cranially to the previous surgical site, while the proximal tip of the shunt catheter remained at the correct location (pink dashed line and arrow) with the resolution of the previously diagnosed SM and SAD

The dog slowly improved and 6 months after shunt placement, MRI was performed: Shunt catheters were in situ, SAD and SM appeared to be resolved, and the spinal cord edema was mild being only present dorsally at the level of C4 vertebra. It was noted the recurrence of the SAD on the right side and cranially to the surgical site; however, this was considered minimal and future monitoring was recommended (Figure 3).

The dog continued to improve, and prednisolone was gradually tapered down over 3‐month period to 0.15 mg/Kg every other day. An update from the owner was obtained 2 years after shunt surgery: Prednisolone was tapered with no further neurological deterioration. Ambulation had greatly improved postshunt surgery, displaying only subtle ataxia and hypermetria referable to his thoracic limbs (Video S2). Episodes of face rubbing against a pillow and licking at his right back paw, without the presence of any skin disease, were subsequently reported. Due to financial constraints, further imaging (even with 80% discount) could not be pursued. Hydrotherapy was restarted, and the signs appeared to subside. No further medical treatment was necessary.

## 3 DISCUSSION

The recognition and diagnosis of SAD associated with SM are increasing in veterinary medicine.^4^, ^5^, ^6^, ^7^, ^8^, ^9^

Spinal arachnoid diverticulum is developmental or acquired intradural‐extramedullary cavities filled with CSF, causing focal compression of the spinal cord parenchyma with secondary myelopathy.^5^ SM is a well‐defined discrete cavity within the spinal cord that contains fluid that is identical or similar to CSF.^10^ However, the exact pathogenesis of these lesions remains still unknown,^9^, ^11^ but it is associated with CSF channel obstruction and in particular Chiari‐like malformation (CM).^6^, ^12^, ^13^

Although our case had other intracranial congenital or developmental malformations such as ventriculomegaly and supracollicular fluid accumulation, CM or other craniocervical junction abnormalities typically associated with SM and SAD ^6^, ^9^ were not present. Furthermore, no infection or previous trauma was reported. This would suggest that the dog's neurological signs associated with partial obstruction of the CSF flow were due to a developmental SAD, causing spinal cord compression and secondary edema especially as the SM was immediate caudal to the SAD. In addition, the reported hyperaesthesia such as face rubbing could be associated with the cervical spinal lesions and greater occipital nerve root irritation (ie, dorsal branch of the second cervical nerve), which is a renowned nerve associated with facial neuralgia.^14^ This appeared to respond to gabapentin which is a drug commonly used to treat neuropathic pain.^15^

Syringomyelia was initially considered to be secondary to SAD, due to changes in temporal CSF pulse pressure dynamics.^6^, ^7^, ^9^, ^11^, ^13^, ^16^, ^17^, ^18^ Therefore, the surgical treatment of SAD via durotomy and marsupialization was considered sufficient to re‐establish normal CSF flow with resolution of SM. Although marsupialization is recommended because of the apparent trend toward a better outcome,^5^ in our case, the resolution of the SM did not occur following surgery. Furthermore, a newly SAD was identified caudal to the previous surgical site and dorsal to SM, with concurrent and progressive signs of CSF disruption and secondary spinal cord edema (Table 1). We therefore opted to use a shunt procedure to treat both conditions. This belt and braces approach was considered safer than another marsupialization and then another surgery to address SM, if that failed. Shunt surgery has been suggested to be beneficial to treat SM,^5^, ^13^ SAD,^6^, ^19^ and recurrent SAD ^20^ or to increase the success rate when an adequate result has not been obtained otherwise.^18^ The cause of SAD recurrence at a more caudal location would suggest the formation of postoperative arachnoid adhesions. Addressing the newly formed CSF‐filled structure with another marsupialization could have led to the creation of other CSF‐filled structures due to the inevitable scar formation, with SM remaining untreated. However, SAD recurrence could have also been related to the natural progression of the disease. The use of a syringopleural shunt in our case appeared more indicated, and it led to the resolution of both SAD and SM. We opted to shunt into the pleural space rather than peritoneal space, due to the limitation in length of the shunt catheter after purse‐string sutures were applied. A syringosubarachnoid or subarachnoid‐subarachnoid shunt placement was not performed, due to the lack of adequate catheter size for the SM size and subarachnoid space available. Moreover, syringopleural shunting would be considered usual practice in humans, as the negative pressure of the intrathoracic cavity appears to be effective for draining syrinx fluid.^21^ Major complications related to shunt placement in dogs are shunt obstruction, pain, shunt infection, catheter migration, and kinking.^22^, ^23^

The shunt catheters used in the previous reports were from either a ventriculoperitoneal shunt kit,^13^, ^19^ Kendall red rubber latex urethral catheter,^19^ equine ocular lavage silicone catheter,^6^ or Silastic™ tube.^5^ Previous studies reported a subarachnoid‐subarachnoid shunt placement to treat SAD,^19^ a syringosubarachnoid shunt placement to treat SM,^6^, ^13^ and a syrinx to the cisterna magna shunt placement to treat SM concurrent to SAD.^5^ In Motta and others (2012), the shunt catheter was not secured to the meningeal layer. In these previous reports, the lack of follow‐up imaging was the major limitation: Only in Motta and others (2012), 3/11 dogs had postsurgical MRI (immediately postoperatively in 1 dog, four weeks after surgery in 1 dog, and six weeks after surgery in 1 dog), while the long‐term follow‐ups were otherwise based on owner's update (via telephone conversations or videos) or physical examination in the others.

In our study, we have performed postoperative imaging up to 1y 2m from the marsupialization and 6m from the shunt placement. Neurologic examinations, telephone, and email updates were obtained up to 2y8m from the marsupialization and 2y from the shunt placement (Table 1). We were able to verify the correct shunt catheter placement, address complications (such as due to shunt catheter displacement), and monitor the disease progress. We have also used a dedicated spinal shunt catheter to divert CSF from the SAD and SM to the pleural space, contrary to the previous reports.^5^, ^6^, ^13^, ^19^ Contrary to the other studies,^7^, ^11^, ^16^, ^19^, ^20^ SM did not resolve following surgery to address SAD, but did resolve following syringopleural shunt surgery and allowed the discontinuation of prednisolone therapy (Table 1). The use of quad wheelchair may have further assisted the dog's rehabilitation and supported his recovery.

Despite the efforts in obtaining postoperative imaging, financial constraints did not allow us to repeat advanced imaging evaluation in the most recent follow‐ups. This remains the major limitation of this study. Hence, we could not further assess the progression of the newly formed SAD.

Finally, the results of our study suggested that marsupialization of SAD did not resolve SM and both conditions needed to be addressed. The placement of a syringopleural shunt allowed us to address both conditions and improve the neurological status allowing discontinuation of corticosteroid therapy. The use of quad wheelchair for dogs can be beneficial to provide further support during postoperative rehabilitation. This study also showed that repeating imaging is essential to confirm the resolution of SAD and SM, the correct shunt placement, identify the reason for neurological deterioration, and choose the most appropriate intervention for our patients.

## CONFLICT OF INTEREST

None declared.

## AUTHOR CONTRIBUTIONS

AT: has made substantial contributions to conception and design, or acquisition of data, or analysis and interpretation of data; has been involved in drafting the manuscript or revising it critically for important intellectual content, and given final approval of the version to be published; and agreed to be accountable for all aspects of the work in ensuring that questions related to the accuracy or integrity of any part of the work are appropriately investigated and resolved. CR: has been involved in the analysis and interpretation of data, and revising it critically for important intellectual content, and given final approval of the version to be published; and agreed to be accountable for all aspects of the work in ensuring that questions related to the accuracy or integrity of any part of the work are appropriately investigated and resolved.

## Supporting information

Video S1

Video S2

## Data Availability

The authors confirm that the data supporting the findings of this study are available within the article and its supplementary materials.
